# Transcriptomic profiling of high- and low-spiking regions reveals novel epileptogenic mechanisms in focal cortical dysplasia type II patients

**DOI:** 10.1186/s13041-021-00832-4

**Published:** 2021-07-23

**Authors:** Arpna Srivastava, Krishan Kumar, Jyotirmoy Banerjee, Manjari Tripathi, Vivek Dubey, Devina Sharma, Nitin Yadav, M. C. Sharma, Sanjeev Lalwani, Ramesh Doddamani, P. Sarat Chandra, Aparna Banerjee Dixit

**Affiliations:** 1 Department of Neurosurgery, AIIMS, New Delhi, 110029 India; 2 Dr B R Ambedkar Centre for Biomedical Research, University of Delhi, Delhi, 110007 India; 3 Department of Biophysics, AIIMS, New Delhi, India; 4 Department of Neurology, AIIMS, New Delhi, India; 5 Department of Pathology, AIIMS, New Delhi, India; 6 Department of Forensic Medicine and Toxicology, AIIMS, New Delhi, India

**Keywords:** Focal cortical dysplasia, Drug-resistant epilepsy, High spiking region, Low spiking region, RNA sequencing, Differential expression

## Abstract

Focal cortical dysplasia (FCD) is a malformation of the cerebral cortex with poorly-defined epileptogenic zones (EZs), and poor surgical outcome in FCD is associated with inaccurate localization of the EZ. Hence, identifying novel epileptogenic markers to aid in the localization of EZ in patients with FCD is very much needed. High-throughput gene expression studies of FCD samples have the potential to uncover molecular changes underlying the epileptogenic process and identify novel markers for delineating the EZ. For this purpose, we, for the first time performed RNA sequencing of surgically resected paired tissue samples obtained from electrocorticographically graded high (MAX) and low spiking (MIN) regions of FCD type II patients and autopsy controls. We identified significant changes in the MAX samples of the FCD type II patients when compared to non-epileptic controls, but not in the case of MIN samples. We found significant enrichment for myelination, oligodendrocyte development and differentiation, neuronal and axon ensheathment, phospholipid metabolism, cell adhesion and cytoskeleton, semaphorins, and ion channels in the MAX region. Through the integration of both MAX vs non-epileptic control and MAX vs MIN RNA sequencing (RNA Seq) data, *PLP1, PLLP, UGT8, KLK6, SOX10, MOG, MAG, MOBP, ANLN, ERMN, SPP1, CLDN11, TNC, GPR37, SLC12A2, ABCA2, ABCA8, ASPA, P2RX7, CERS2, MAP4K4, TF, CTGF, Semaphorins, Opalin, FGFs, CALB2,* and *TNC* were identified as potential key regulators of multiple pathways related to FCD type II pathology. We have identified novel epileptogenic marker elements that may contribute to epileptogenicity in patients with FCD and could be possible markers for the localization of EZ.

**Supplementary Information:**

The online version contains supplementary material available at 10.1186/s13041-021-00832-4.

## Introduction

Focal cortical dysplasia is the most commonly encountered developmental malformation that causes drug resistant focal epilepsy, particularly in children [1]. Its anatomopathological position and cellular appearance are highly variable and influence not only the cortical architecture and unique neuronal subpopulations, but also the junction of gray-white matter and sub-cortical white matter regions [2, 3]. The most frequent subtype is FCD type II, mainly localized in the frontal and parietal lobes and can range from either small and almost invisible bottom‐of‐sulcus dysplasia to larger dysplastic regions covering more than a single gyrus. Focal cortical dysplasia type II is marked by gross histopathological changes, i.e., dysmorphic neurons (FCD type IIA) and additional balloon cells (FCD type IIB) [4, 5]. Because dysplastic tissue contains atypical neuronal networks that are highly susceptible to abnormal excitation, FCD is thought to be intrinsically epileptogenic. Despite the introduction of new anti-epileptic drugs (AEDs) in the last two decades, over 30% of epilepsy patients have recurring seizures and many have undesirable side effects. Surgery is an effective alternative treatment as it offers seizure freedom or a significant reduction in seizures for those patients with drug-resistant epilepsy (DRE). Epilepsy surgical outcome is influenced by a number of factors, including epilepsy type, underlying pathology, and the most significant accurate localization of the epileptogenic zone (EZ) and precise details of its association with the eloquent cortex for complete and safe removal using a variety of clinical, neuroimaging, and neurophysiological tests [6–8].

FCD is a diffuse lesion with poorly defined epileptogenic zones. Thus, incomplete resection has been consistently known to be a poor prognostic factor. Clinical history, comprehensive semiology analysis, long-term video-EEG recording, inter-ictal and ictal EEG analysis, neuroimaging, and neuropsychological examination are all part of the pre-surgical evaluation process and each modality gives distinct and complementary information. Because no single currently available approach can consistently diagnose EZ, and each modality has its own set of limitations, comprehensive examinations are required to analyse the EZ's various characteristics [8, 9]. The functional involvement of the dysplastic cortex in the epileptogenic network cannot be identified through MRI alone. FCDs can be microscopic (or MRI negative), which means they may go undetected even with high resolution MRI. The lesions are subtle in these cases; morphological features may vary only marginally from normal tissue [10, 11]. fMRI or magnetoencephalography (MEG) detects classical and aberrant distributed functional networks but may be falsely suppressed in the postictal period. The absence of a visible lesion is one of the greatest challenges in epilepsy surgery; dysplastic tissue looks similar to normal brain tissue and can be missed, unless intracranial electrode application and intraoperative electrocorticography (EcoG) recordings are performed [6, 12].

Despite the use of all available invasive and non-invasive approaches, the epileptogenic zone cannot be fully identified, and patients do not benefit in more than 30% of these cases, owing to the inability to accurately locate the EZ [13]. A more precise framework for identifying EZ can be provided by molecular and cellular biomarkers combined with imaging and electrical investigations [13, 14]. Aberrant gene expression and epigenetic alterations such as DNA methylation have been reported in different epilepsy pathologies, including FCD [15–20]. These studies have helped us to better understand the molecular mechanism of epileptogenesis, but the search for biomarkers to localize the EZ accurately has not ended yet.

This has kindled interest in unveiling the mechanisms of epileptogenicity in these lesions. Human tissue samples, on the other hand, restrict experimental design because age and gender-matched control samples from non-epileptic patients are rarely available for comparison. However, having a better understanding of how seizures are generated in the dysplastic human neocortex ultimately requires an examination of the available human tissue samples. Surgically resected human dysplastic tissue can be a good model to study the mechanism of epileptogenicity in these patients. Intracranial EcoG recording is usually performed in FCD cases to identify the extent of the epileptogenic zone and its complete excision. Tissues with different EcoG grades have been removed during surgery from the same patients. These tissues from the same patient can be an ideal model to extrapolate the mechanism of epiletogenicity in FCD type II, which in turn helps to delineate the epileptozenic zone in FCD patients. Hence, the present study was designed to study the transcriptomic profile of surgically resected paired tissue samples obtained from electrocorticographically graded high- (MAX) and low-spiking (MIN) regions of FCD type II patients and autopsy controls. The current study’s findings are discussed to gain better understanding of the epileptogenicity in FCD and the localization of EZ.

## Materials and methods

### Patient and control samples

The patients who were diagnosed to have DRE due to FCD type II and underwent electrocorticography (ECoG)-guided surgery were included in the study. Pre-surgical assessment was done for each patient, and the pathology was determined by analysing convergent data on MRI, video EEG (vEEG), fluoro-2-deoxyglucose positron emission tomography (FDG-PET) and magnetoencephalography (MEG), further confirmed by histopathological examinations by neuropathologists. Patients with dual pathology were excluded.

Based on ECoG recordings, the regions were graded from scores of 1 to 5 [21, 22], with grade 2 and above reported as a high spiking zone (MAX) and grade 1 as a low spiking zone (MIN) (Additional file 1: Fig. S1). Surgical resection of ECoG graded cortical samples was performed as per the previously reported protocol [22, 23]. The MAX region was defined as cortical regions of MEG abnormality, the greatest positron emission tomography hypometabolism, the most severe magnetic resonance imaging architectural abnormalities, and the most abnormal ECoG findings. The MIN region was defined as less severely involved based on neuroimaging and ECoG, but it was part of the planned resection. Resected tissue samples from the MAX and MIN regions from the same patients were collected for transcriptomic analysis. Details of neuroimaging techniques used and their scores are listed in Table 1. Based on EcoG grade, MRI, PET and MEG data, E018, E019, E070, E075, E077, E273, E460, and E578 were categorized under the MIN region, and E006, E028, E045, E084, SampleE1, E115, E135, E536 and E593 were categorized under the MAX region.

**Table1:** Clinical characteristics of patients and controls

S. no.	Patient ID	Sample ID	Age (years)	Sex	Pathology/COD	ECOG	MRI	MEG	PET	Age at onset	Frequency of seizure	Anti-epileptic drugs
1	F1	E018	13	F	FCD TYPE IIB	1	0	ND	0	6 years	3–4/day	CLM, LVM, SV
2	F1	E115				4	0	ND	0			
3	F2	E075	18	M	FCD TYPE IIB	1	0	1	0	1.5 years	1–2/day	LMG, CLM, LVM, TPM
4	F2	E028				3	1	0	1			
5	F3	Sample-E1	24	F	FCD TYPE IIA	5	0	0	0	8 months	1–2/day	CLM, LCS, OXCBZ, SV
6	F4	E019	6	M	FCD TYPE IIA	1	0	0	1	3 years	6–7/day	LVM, CBZ, CLM
7	F4	E006				5	1	0	1			
8	F5	E273	5	F	FCD TYPE IIB	1	0	1	0	2 months	1–2/day	CLM, OXCBZ
9	F5	E135				4	1	1	1			
10	F6	E077	14	M	FCD TYPE IIA	1	1	ND	0	9 years	2–3/month	CLM, LVM, PHY
11	F6	E045				3	1	ND	1			
12	F7	E070	12	F	FCD TYPE IIB	1	0	0	0	5 years	5–7/day	LVM, OXCBZ, CLM
13	F7	E084				5	1	0	1			
14	F8	E460	19	F	FCD TYPE IIA	1	0	1	0	9 months	2–3/day	LMG, CLM
15	F8	E536				5	0	1	1			
16	F9	E578	22	F	FCD TYPE IIA	1	0	1	0	6 years	3–4/day	LVM, CBZ, TPM, CLM
17	F9	E593				3	1	1	0			
18	A1	A1	18	F	Pelvic injury	NA	NA	NA	NA	NA	NA	NA
19	A2	A2	25	M	Abdominal injury	NA	NA	NA	NA	NA	NA	NA
20	A3	A3	22	M	Pelvic and limb injury	NA	NA	NA	NA	NA	NA	NA
21	A4	A4	37	M	Abdominal injury	NA	NA	NA	NA	NA	NA	NA
22	A5	A5	32	M	Abdominal injuries	NA	NA	NA	NA	NA	NA	NA
23	A6	A6	20	M	Hanging	NA	NA	NA	NA	NA	NA	NA
24	A7	A7	65	F	Myocardial infarction	NA	NA	NA	NA	NA	NA	NA
25	A8	A8	16	F	Pelvic and limb injury	NA	NA	NA	NA	NA	NA	NA

*COD*  cause of death, *ND*  not done, *0*  Negative, *1*  Positive, *CBZ* carbamazepine, *OXCBZ*  oxcarbazepine, *CLM*  clobazam, *SV*  sodium valproate, *PHE*  phenytoin, *LVM*  leveteracetam, *LMG*  lamotrigine, *TP*  topiramate

As there are no “ideal” or acceptable non-epilepsy controls for such studies involving epilepsy surgery, we have used histologically normal cortex tissues obtained from the frontal lobes of the post-mortem cases without any history of seizures or other neurological disorders as non-epileptic controls. All the autopsies were performed within 8 h of death. All the patients included in the study were seizure-free post-operatively (Class I Engel outcome). Part of the resected tissue were stored in 4% paraformaldehyde for histopathological examination and remaining parts were immediately frozen and stored at − 80 °C until further use.

The study was conducted in accordance with the Declaration of Helsinki and was approved by the Institute Ethics Committee, AIIMS, New Delhi. Informed and written consent was obtained from all the patients, their parents, or legal guardians if the patients were underage.

### RNA sequencing (RNA seq)

RNA extraction and sequencing were performed as described previously with some modifications [16]. Briefly, frozen brain samples were homogenized in RiboZol reagent (Amresco) and RNA was extracted using RNeasy Mini Kit (Qiagen) as per manufacturer’s instructions. An additional DNase1 digestion step was performed to ensure that the samples were not contaminated with genomic DNA. RNA quality was assessed using Bioanalyzer 2100 (Agilent). RNA libraries were prepared using TruSeq RNA Access Library Prep Kit (Illumina) and paired-end sequencing was performed on IlluminaHiseq 2500 platforms. Sequences were quality-checked using FastQC and low-quality bases and reads were excluded from further analysis. Sequences were aligned using HISATaligner against all known genes and transcripts of GRCh37/hg19 assembly.

In this comparative study we analyzed the RNA Sequencing data by three of the most frequently used software tools: Cuffdiff, DESeq2 and EdgeR [24, 25]. Significantly altered genes which were common in all three software tools were further used for downstream gene enrichment and network analysis. RNA Seq analysis was performed for three categories: (1) Between autopsy samples (A1 andA2) and samples from MIN region (E018, E019, E075 and E273); (2) Between autopsy samples (A1 andA2) and samples from MAX region (E006, E028, E115, SampleE1 and E135); and (3) Between samples from MIN region (E018, E019, E075 and E273) and samples from MAX region (E006, E028, E115, SampleE1, and E135).

### Principal component analysis (PCA), pathway enrichment analysis and gene network analysis

Intersections of gene expression which were found to be significantly altered in all three RNA Seq analysis software were used for calculating and plotting the principal components using ClustVis [26]. Unit variance scaling was applied to genes and singular value decomposition with imputation was used to calculate the principal components. Samples were clustered using correlation distance and average linkage in heatmap. Common DEGs (based on fold-change (≥ 2) and FDR-adjusted p values (padj) in all three software packages were used for downstream gene enrichment and network analyses. Gene enrichment analysis was performed as described previously [27]. Briefly, the DEGs were used as an ordered query in g: Profiler with term size ranging from 3 to 350 and significance cut-off (FDR q val) set to < 0.05. Custom gene sets containing GO: BP terms and KEGG pathways were used.

Network analysis was performed to graphically display associations between DEGs, to show both direct and indirect interactions using Natural language processing-based (NLP) network discovery algorithms in gene spring software version 13.0 as described previously [16].

### Validation by real-time PCR (RT-PCR)

Real-time PCR (RT-PCR) was performed to validate the differentially expressed genes using specific primers (Additional file 7: Table S1) for selected genes (*TNC, MOBP, SLC12A2, CTGF, GPR37, KCNK10* and *CARTPT*) on 9 FCD (F1 to F9) patients and 8 control samples (A1 to A8). RNA was reverse transcribed using High-capacity cDNA Reverse Transcription Kit (ThermoFisher; catalogue# 4368814). Hypoxanthine phosphoribosyltransferase (*HPRT*) gene was used as an internal reference. Real-time PCR amplifications were performed in CFX 96 real-time systems (Bio-Rad) with Bio-Rad CFX software manager with the following cycling parameters: an initial hot start of 95 °C for 3 min followed by 40 cycles of 95 °C for 5 s and 60 °C for 30 s. The melting curve of each replicate was examined to confirm a single peak appearance. All samples were run in triplicates. The 2^−ΔΔCq^ method was used to measure the mRNA expression of target genes based on the average Ct values across samples [28].

### Histopathology

Tissues were fixed in 4% paraformaldehyde and embedded in paraffin wax for preparing 5-µm thick tissue sections. Haematoxylin and eosin (H&E) staining was performed as described previously [16]. One series of sections was stained with crystal violet (Sigma) according to the previously established protocol [29]. The slides were independently reviewed by two neuropathologists to confirm the pathology and evaluate any damage in control tissue.

## Results

### Clinical characteristics of patients and controls

A total of nine FCD Type II patients (three male and six females) patients were included in this study. For RNA Seq analysis, graded samples of five FCD type II patients (F1 to F5) and two controls (A1 to A2) were included. Subsequently, we used surgically resected graded tissues from 9 FCD type II patients (F1–F9) and eight controls (A1–A8) (including samples of RNA Seq analysis) for real-time PCR analysis. The detailed clinical characteristics of individuals are listed in Table 1.

**Fig. 1:**
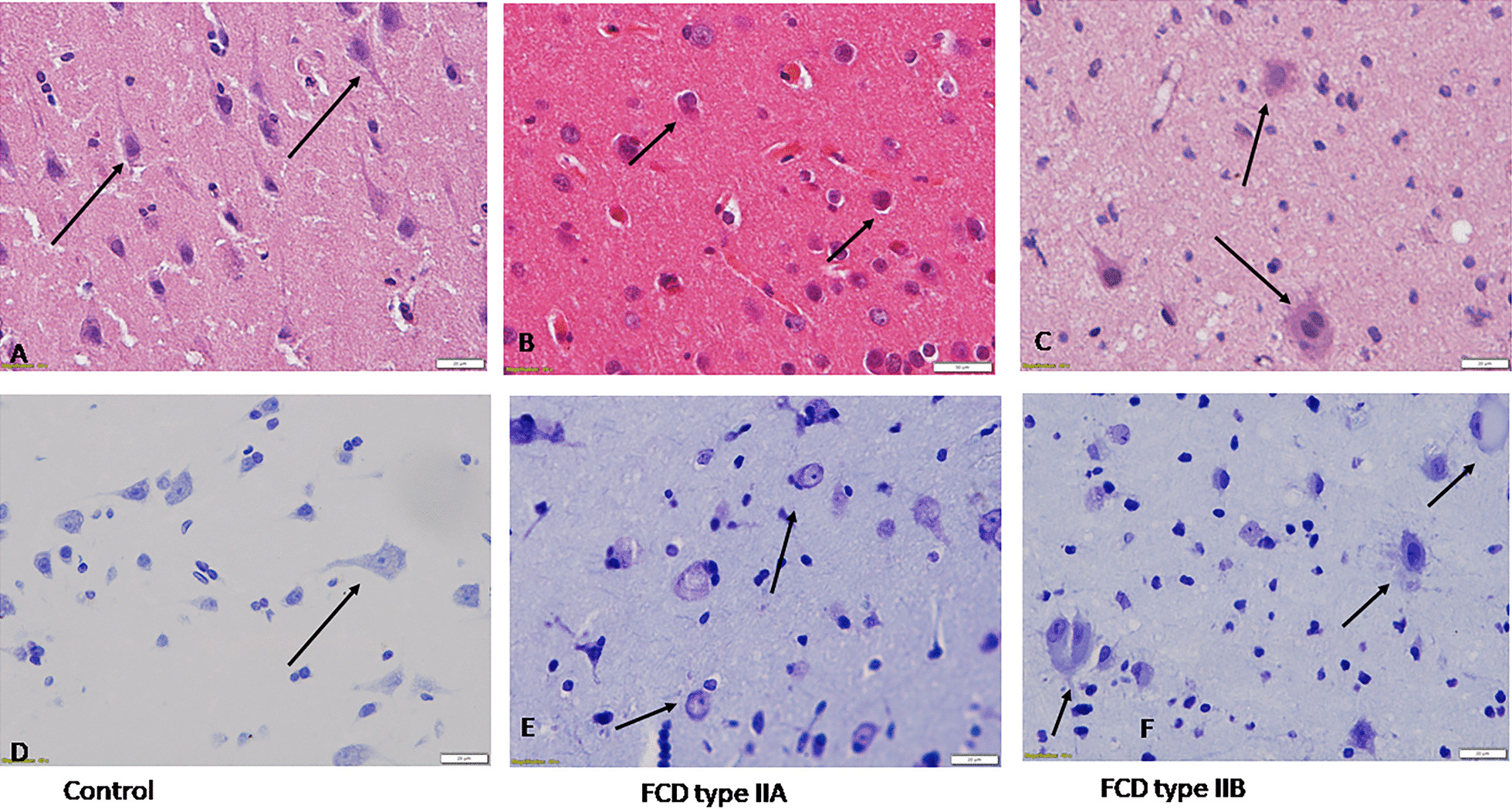
Photomicrograph showing characteristic histopathological features of focal cortical dysplasia II. Representative image of the cortical section from FCD type II patients showing dysmorphic neurons **B** HE, × 400) and **E** CV staining × 400; and dysmorphic neurons with balloon cells **C** HE, × 400) and **F** CV staining × 400. Cortical sections from autopsy showed normal cytoarchitechure. **A** HE, × 400 and **D** CV staining × 400

The mean age of FCD type II patients was 14.77 ± 6.23 years (ranges from 5 to 22 years). Autopsy patient’s age ranged from 16 to 65 years (mean age 29.37 ±  15.01 years). Detailed histopathological investigations were performed on all the samples obtained for experiments (as mentioned in Table 1) to confirm the pathology (Fig. 1). Haematoxylin and eosin (HE), and crystal violet (CV) staining were performed to evaluate the histopathological features. Characteristic features of FCD type II patients were observed in all the patients. Cortical section from FCD type II patients showed dysmorphic neurons Fig. 1B, E and dysmorphic neurons with balloon cells Fig. 1C, F. Cortical sections from autopsy showed normal cytoarchitecture Fig. 1A, D.

### Differentially expressed genes (DEGs)

**Fig. 2:**
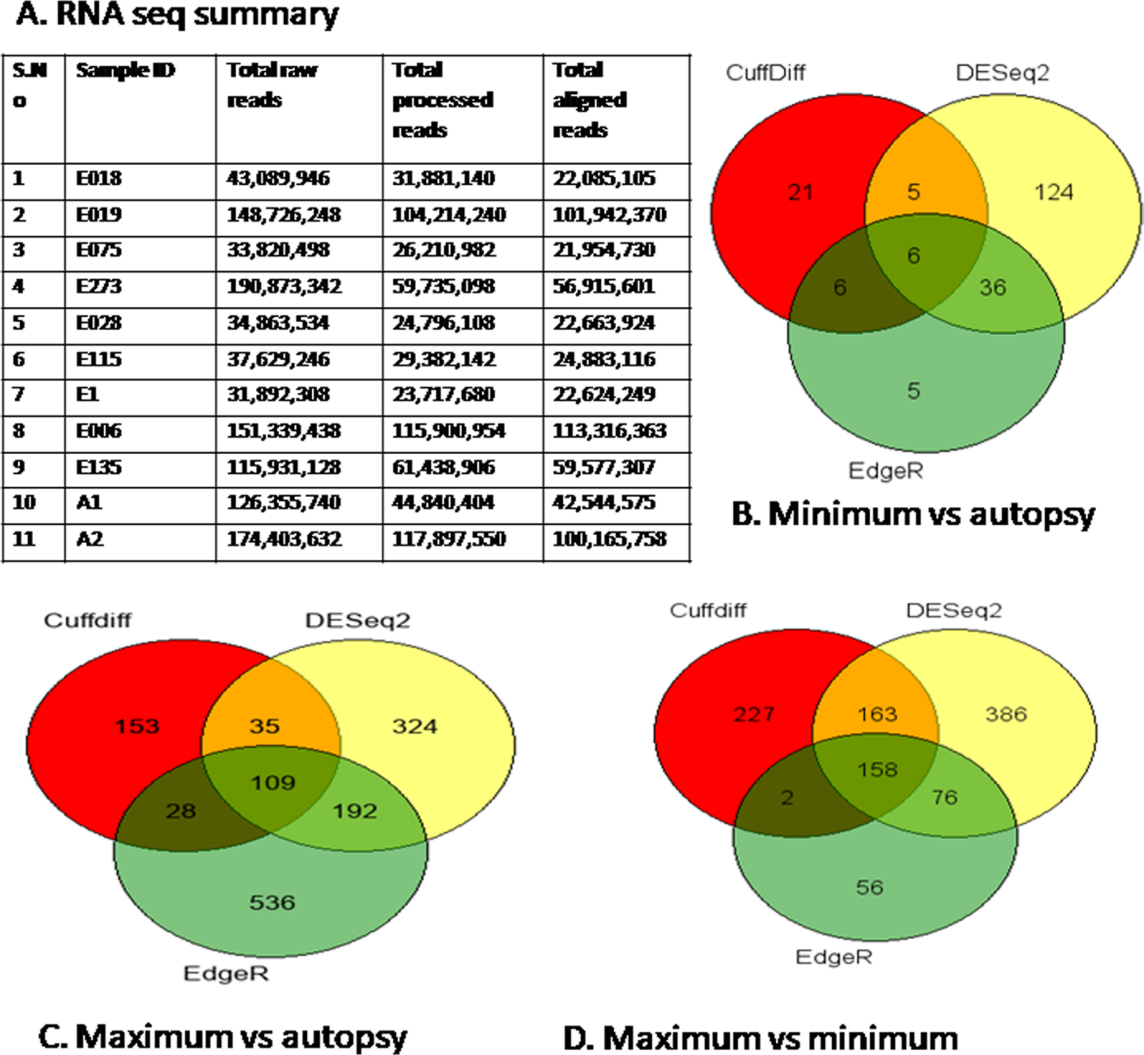
**A** RNA Seq summary of samples from patients and controls **B**, **C** and **D**. The Venn diagram depicts both unique and common DEGs identified by three softwares: CuffDiff, DESeq2 and EdgeR in three groups: MIN vs autopsy (**B**), MAX vs autopsy (**C**), and MAX vs MIN (**D**)

**Fig. 3:**
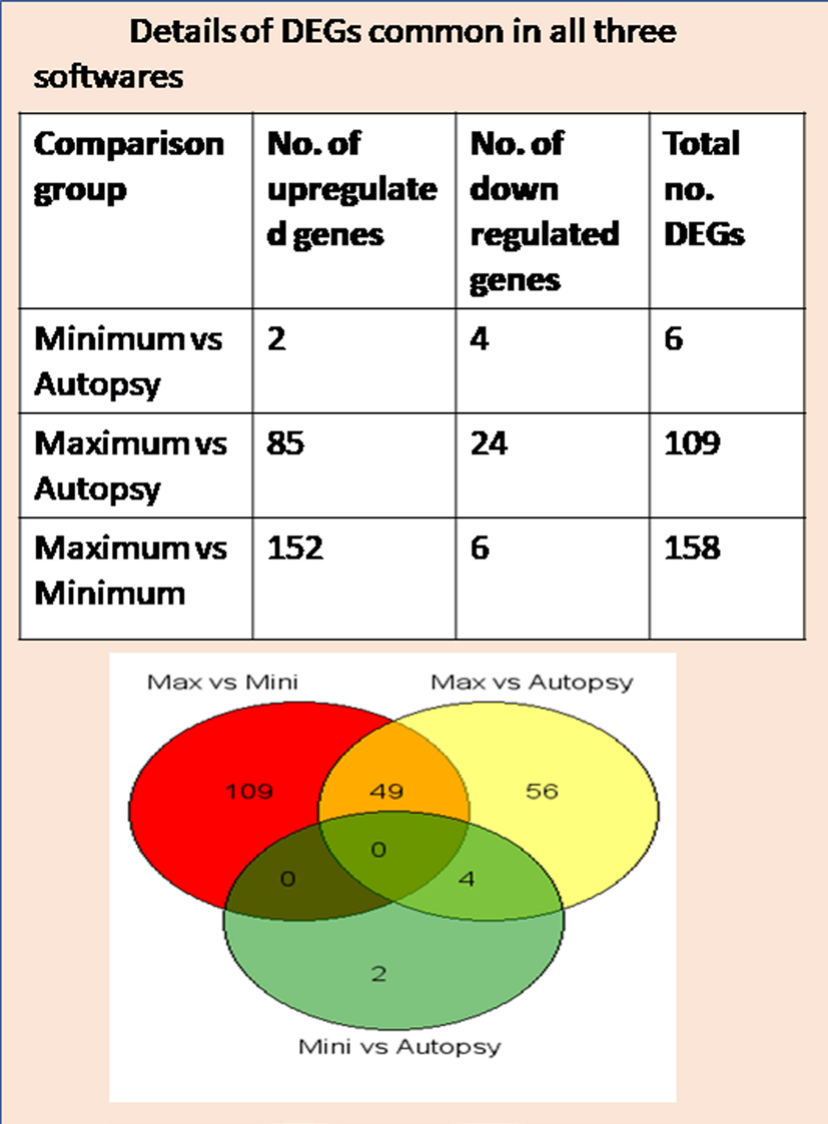
Details of differentially expressed genes in all three groups (MIN vs autopsy, MAX vs autopsy, and MAX vs MIN), were found to be common in CuffDiff, DESeq2, and EdgeR analysis software

RNAseq read summary is provided in the Fig. 2A. Cuffdiff analysis revealed 38 differentially expressed genes (16 up-regulated and 22 down-regulated) in MIN vs autopsy; 325 DEGs (224 up-regulated and 101 down-regulated) in MAX vs autopsy; and 550 DEGs (378 up-regulated and 172 down-regulated) in MAX vs MIN (Fig. 2B–D; Additional file 3). List of genes with significantly altered expression analysed by Cuffdiff is provided in the Additional file 3 As per DESeq2 analysis, a total of 171 genes were found to be differentially expressed (56 up-regulated and 115 down-regulated) in MIN vs autopsy; 660 genes (316 up-regulated and 344 down-regulated) in MAX vs autopsy; and 783 genes (582 up-regulated and 201 down-regulated) in MAX vs MIN (Fig. 2B–D; Additional file 4). List of DEGs analysed by DESeq2 is given in Additional file 4. EdgeR analysis demonstrated 53 DEGs (5 up-regulated and 48 down-regulated) in MIN vs autopsy; 865 DEGs (176 up-regulated and 689 down-regulated) in MAX vs autopsy; and 292 DEGs (240 up-regulated and 52 down-regulated) in MAX vs MIN (Fig. 2B–D). List of genes with significantly altered gene expression analysed by EdgeR is provided in Additional file 5. Most of the DEGs identified by each of three tools overlapped, DESeq2 detected more DEGs than the other tools. To avoid false positive results, intersection of DEGs from two or more tools is generally used for analysis [24, 25]. To get more accurate and precise findings, intersection of DEGs from Cuffdiff, DESeq2 and EdgeR was used for further analysis, details of commonly found DEGs among three tools is presented in Fig. 3 and Additional file 6. Only 6 genes (2 up-regulated and 4 down-regulated) were found to be differentially expressed in MIN vs autopsy, 109 DEGs (85 up- regulated and 24 down-regulated) were observed in MAX vs autopsy, and 158 DEGs (152 up-regulated and 6 down-regulated) were found to be significantly altered in MAX vs MIN. No gene was found to be common in all three groups. 49 genes were found to be common in MAX vs autopsy and MAX vs MIN. 4 genes were observed to be common in MIN vs autopsy and MAX vs autopsy (Fig. 3).

**Fig. 4:**
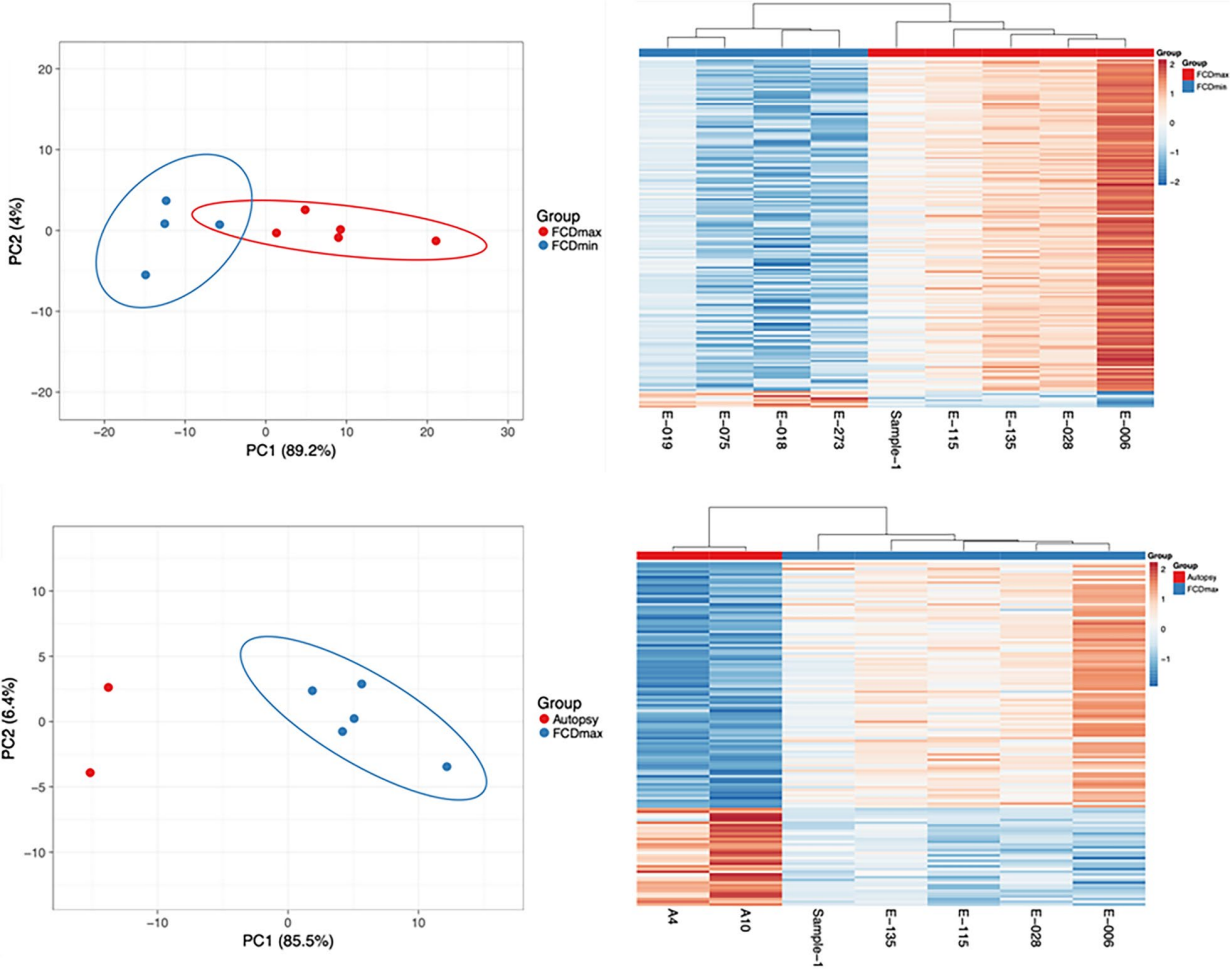
Differential gene expression in FCD type II patients. The PCA analysis and heatmap was generated using ClustVis. For Peer Review Singular value decomposition with imputation was used to calculate the PCs with maximum variance explained by PC1 (89.2% and 85.5% respectively). Plotting of the first two PCs (PC1 and PC2) segregated FCD MAX and control samples; and FCD MIN and FCD MAX samples into distinct clusters. Unit variance scaling is applied to rows containing the log-normalized counts of differentially expressed genes (padj1) and the columns are clustered using correlation distance and average linkage in the heatmap

The PCA result indicated that MIN and MAX region of FCD type II patients could be separated by their transcriptome profile by unsupervised clustering. Dimensionality reduction using principal component analysis segregated FCD type II samples and autopsy samples into distinct clusters with PC1 (85.5% for autopsy and MAX, and 89.2% for MIN and MAX) accounting for most of the variance (Fig. 4).

### Pathway enrichment analysis and network analysis

Detailed pathway g: Profiler enrichment results are provided in the Table 2 (FDR q value < 0.05). Pathway enrichment did not provide any result for MIN vs autopsy. Pathway enrichment scores revealed enrichment of altered genes in the four pathways related to myelination, oligodendrocytes development and neuronal and axon ensheathment in MAX vs autopsy, represented by *PLP1, MAG, UGT8, CD9, PLLP, SH3TC2*. Myelination, ensheathment of neurons and axons, oligodendrocyte development and differentiation, gliogenesis, glial cell development and differentiation, phospholipid biosynthesis, cell adhesion and cytoskeleton proteins, neurogenesis, nervous system development, small molecule transport, and ion channels are among the 44 significantly enriched pathways for MAX vs MIN. Genes related to myelination, oligodendrocytes development and differentiation, neuronal and axon ensheathment included *PLP1, SOX10, MAG, MOG, MOBP, KLK6, UGT8, CLDN11, ASPA, SH3TC2, NKX6-2, FA2H,CTGF, SEPT 4, CDKN1C, GLDN, SPP1, CNTN2, TMEM98, TMEM10 (OPALIN), ANLN, ERMN, ENPP2, CD9, ABCA2, ABCA8, SPP1, GPR37* and *TF*. All these were found to be up-regulated. Among these 9 genes (*KLK6, ASPA, SOX10, CNTN2, CLDN11, ERMN, NKX6-2, FA2H, SEPT4, SPP1, MOG, NKX6-2* and *MOBP*) were found to be significantly up-regulated only in MAX vs MIN, whereas *CTGF* was found to be up-regulated only in MAX vs autopsy. *PLLP, UGT8, ABCA2, PLD1, ELOVL1, CERS2, S1PR5, PLPPR1, SPTLC1, ENPP2, ENPP4, ENPP6, NPC1, FA2H, LRP2, P2RX7, S1PR5 and GAL3ST1* were reported to be involved in phospholipid biosynthesis. Of these, *ABCA2, PLD1, ELOVL1, FA2H, CERS2, P2RX7,* and *GAL3ST1* were found to be up-regulated in MAX vs MIN only, whereas expression of *SPTLC1* was observed to be up-regulated only in MAX vs autopsy. Genes related to ion and water channels i.e., *SLC12A2, SLC45A3, SLC26A9, SLC44A1, SLC26A9, AQP1, KCNK10, KCNH8, P2RX7, SGK1, SGK2,* and *SLC6A2* were found to be up-regulated. Among these, *SLC12A2, SLC45A3, SGK1* and *SLC25A41* were found to be up-regulated only in MAX vs autopsy, however, *AQP1, P2RX7, SLC6A2, SLC26A9, SLC44A1, SLC45A3, SLC5A11* and *SLC26A9* were found to be up-regulated only in MAX vs MIN. Semaphorins (*SEMA4D, SEMA3B* and *SEMA6A*) were found be up-regulated only in MAX vs MIN. Cell signaling molecules of various functions, *MAP4K4, TNC, FGF1, FGF17, TGFA, ATF3, MCAM, TGFBRII* were found to be up-regulated, *GRP* and *CALB2* were found to be down-regulated. Of these, *FGF1, MAP4K4, ATF3, CALB2* and *GRP* were significantly altered only in MAX vs MIN, whereas expression of *FGF17* and *TGFBR2* were significantly up-regulated only in MAX vs autopsy. Extracellular matrix (ECM) related genes *TJP2, CLDN9, CLDN11, SPP1,* and *GJB1* were also found to be up-regulated. *TJP2, SPP1, CLDN11* were found to be up-regulated only in MAX vs MIN whereas expression of CLDN9 was up-regulated in only MAX vs autopsy.

**Table2:** Pathway enrichment analysis for DEGs

Pathway enrichment analysis									
Source	Term_name	Term_id	Adjusted_p_value	Negative_log10_of_adjusted_p_value	Term_size	Query_size	Intersection_size	Effective_domain_size	Intersections
*A: Maximum vs autopsy*									
GO:BP	Myelination	GO:0042552	0.00168791	2.77265077	139	43	6	17906	PLP1, MAG, UGT8, CD9, PLLP, SH3TC2
GO:BP	Ensheathment of neurons	GO:0007272	0.00191291	2.71830651	142	43	6	17906	PLP1, MAG, UGT8, CD9, PLLP, SH3TC2
GO:BP	Axon ensheathment	GO:0008366	0.00191291	2.71830651	142	43	6	17906	PLP1, MAG, UGT8, CD9, PLLP, SH3TC2
GO:BP	Oligodendrocyte development	GO:0014003	0.04500542	1.34673519	47	22	3	17906	PLP1, MAG, CD9
*B: Maximum vs minimum*									
GO:BP	Ensheathment of neurons	GO:0007272	1.44E−11	10.8420434	142	158	17	17906	PLP1,KLK6,UGT8,CLDN11,ASPA,SH3TC2,MAG,NKX6-2,FA2H,CNTN2,TMEM98,CD9,GAL3ST1,ABCA2,PLLP,BCAS1,SOX10
GO:BP	Axon ensheathment	GO:0008366	1.44E−11	10.8420434	142	158	17	17906	PLP1,KLK6,UGT8,CLDN11,ASPA,SH3TC2,MAG,NKX6-2,FA2H,CNTN2,TMEM98,CD9,GAL3ST1,ABCA2,PLLP,BCAS1,SOX10
GO:BP	Myelination	GO:0042552	1.72E−10	9.76340897	139	158	16	17906	PLP1,KLK6,UGT8,ASPA,SH3TC2,MAG,NKX6-2,FA2H,CNTN2,TMEM98,CD9,GAL3ST1,ABCA2,PLLP,BCAS1,SOX10
GO:BP	Oligodendrocyte differentiation	GO:0048709	9.24E−09	8.03431166	101	158	13	17906	SLC45A3,PLP1,ASPA,MAG,NKX6-2,FA2H,CNTN2,TMEM98,CD9,ABCA2,BOK,DAAM2,SOX10
GO:BP	Axon ensheathment in central nervous system	GO:0032291	2.50E−08	7.60287967	23	158	8	17906	PLP1,ASPA,MAG,NKX6-2,FA2H,CNTN2,ABCA2,SOX10
GO:BP	Central nervous system myelination	GO:0022010	2.50E−08	7.60287967	23	158	8	17906	PLP1,ASPA,MAG,NKX6-2,FA2H,CNTN2,ABCA2,SOX10
GO:BP	Oligodendrocyte development	GO:0014003	4.89E−07	6.311047	47	158	9	17906	PLP1,ASPA,MAG,NKX6-2,FA2H,CNTN2,CD9,ABCA2,SOX10
GO:BP	Gliogenesis	GO:0042063	2.13039E−05	4.67154125	310	134	15	17906	LRP2,SLC45A3,PLP1,ASPA,SH3TC2,MAG,NKX6-2,FA2H,CNTN2,TMEM98,CD9,CERS2,ABCA2,BOK,DAAM2
GO:BP	Glial cell differentiation	GO:0010001	2.48143E−05	4.60529884	226	158	14	17906	SLC45A3,PLP1,ASPA,SH3TC2,MAG,NKX6-2,FA2H,CNTN2,TMEM98,CD9,ABCA2,BOK,DAAM2,SOX10
GO:BP	Glial cell development	GO:0021782	9.74491E−05	4.01122223	121	109	9	17906	PLP1,ASPA,SH3TC2,MAG,NKX6-2,FA2H,CNTN2,CD9,ABCA2
GO:BP	Sphingolipid biosynthetic process	GO:0030148	0.000460392	3.33687211	105	109	8	17906	UGT8,ELOVL1,PLPP2,FA2H,P2RX7,CERS2,GAL3ST1,ABCA2
GO:BP	Regulation of gliogenesis	GO:0014013	0.000494076	3.30620614	132	158	10	17906	LRP2,SLC45A3,ASPA,MAG,NKX6-2,CNTN2,TMEM98,CERS2,DAAM2,SOX10
GO:BP	Nervous system development	GO:0007399	0.000672174	3.17251812	2432	160	46	17906	LRP2,AQP1,SLC45A3,MOG,PLP1,KLK6,UGT8,CLDN11,SPP1,GLDN,GJB1,ASPA,MOBP,SH3TC2,C21ORF91,MAG,S1PR5,NKX6-2,FA2H,CNTN2,DOCK10,TMEM98,CD9,USH1C,SEMA4D,TNC,NDE1,CERS2,MYO1D,GAL3ST1,ABCA2,PLLP,ZNF536,SEMA6A,BOK,SYNJ2,SEMA3B,MAN2A1,BCAS1,DAAM2,KIF13B,WHRN,FRYL,SFRP1,SOX10,MAP4K4
GO:BP	Neurogenesis	GO:0022008	0.000902115	3.04473832	1679	166	37	17906	LRP2,SLC45A3,PLP1,KLK6,UGT8,SPP1,GLDN,ASPA,SH3TC2,C21ORF91,MAG,S1PR5,NKX6-2,FA2H,CNTN2,DOCK10,TMEM98,CD9,USH1C,SEMA4D,TNC,NDE1,CERS2,ABCA2,ZNF536,SEMA6A,BOK,SEMA3B,MAN2A1,DAAM2,KIF13B,WHRN,FRYL,SFRP1,SOX10,MAP4K4,LTK
GO:BP	Cell development	GO:0048468	0.000919176	3.03660132	2215	160	43	17906	LRP2,SLC45A3,PLP1,KLK6,UGT8,SPP1,GLDN,ASPA,TYMS,DYSF,SH3TC2,DOCK5,C21ORF91,MAG,S1PR5,LDB3,CAPN3,NKX6-2,FA2H,CNTN2,PIP4K2A,DOCK10,TMEM98,CD9,USH1C,SEMA4D,TNC,CERS2,ABCA2,TJP2,ZNF536,SEMA6A,SEMA3B,MAN2A1,CLIC4,CFL2,DAAM2,KIF13B,WHRN,FRYL,SFRP1,SOX10,MAP4K4
GO:BP	Regulation of glial cell differentiation	GO:0045685	0.001515404	2.81947151	73	79	6	17906	SLC45A3,ASPA,MAG,NKX6-2,CNTN2,TMEM98
GO:BP	Regeneration	GO:0031099	0.003378801	2.4712374	209	96	9	17906	KLK6,SPP1,TYMS,DYSF,MAG,CAPN3,CD9,TNC,CERS2
GO:BP	Ceramide biosynthetic process	GO:0046513	0.00370985	2.4306437	64	105	6	17906	UGT8,ELOVL1,FA2H,P2RX7,CERS2,GAL3ST1
GO:BP	Membrane lipid biosynthetic process	GO:0046467	0.005378894	2.26930705	145	109	8	17906	UGT8,ELOVL1,PLPP2,FA2H,P2RX7,CERS2,GAL3ST1,ABCA2
GO:BP	Negative regulation of neurogenesis	GO:0050768	0.006339891	2.19791821	303	160	13	17906	SPP1,MAG,NKX6-2,CNTN2,TMEM98,SEMA4D,CERS2,ZNF536,SEMA6A,SEMA3B,DAAM2,SOX10,MAP4K4
GO:BP	Galactosylceramide biosynthetic process	GO:0006682	0.007164655	2.14480471	6	105	3	17906	UGT8,FA2H,GAL3ST1
GO:BP	Galactolipid biosynthetic process	GO:0019375	0.007164655	2.14480471	6	105	3	17906	UGT8,FA2H,GAL3ST1
GO:BP	Regulation of neurogenesis	GO:0050767	0.007966259	2.09874557	856	166	23	17906	LRP2,SLC45A3,KLK6,SPP1,ASPA,C21ORF91,MAG,S1PR5,NKX6-2,CNTN2,TMEM98,SEMA4D,CERS2,ZNF536,SEMA6A,SEMA3B,MAN2A1,DAAM2,KIF13B,SFRP1,SOX10,MAP4K4,LTK
GO:BP	Cellular component morphogenesis	GO:0032989	0.010095286	1.99588137	1177	160	27	17906	LRP2,UGT8,ENPP2,SPP1,ERMN,DOCK5,CDH19,MAG,LDB3,CAPN3,CNTN2,P2RX7,DOCK10,CD9,SEMA4D,WIPF1,ABCA2,CDC42EP1,SEMA6A,SEMA3B,PHLDB1,CLIC4,CFL2,KIF13B,WHRN,FRYL,MAP4K4
GO:BP	Sphingolipid metabolic process	GO:0006665	0.011698213	1.93188047	161	109	8	17906	UGT8,ELOVL1,PLPP2,FA2H,P2RX7,CERS2,GAL3ST1,ABCA2
GO:BP	Negative regulation of nervous System development	GO:0051961	0.013118962	1.88210053	324	160	13	17906	SPP1,MAG,NKX6-2,CNTN2,TMEM98,SEMA4D,CERS2,ZNF536,SEMA6A,SEMA3B,DAAM2,SOX10,MAP4K4
GO:BP	Regulation of cell projection organization	GO:0031344	0.015838735	1.8002795	704	166	20	17906	ANLN,KLK6,ENPP2,SPP1,ERMN,C21ORF91,MAG,CNTN2,P2RX7,USH1C,SEMA4D,CERS2,PLD1,CDC42EP1,SEMA6A,SEMA3B,KIF13B,SFRP1,MAP4K4,LTK
GO:BP	System development	GO:0048731	0.018013177	1.74440968	5020	167	73	17906	LRP2,AQP1,SLC45A3,MOG,PLP1,ANLN,DMRT2,PCSK6,KLK6,UGT8,CLDN11,ENPP2,SPP1,GLDN,GJB1,ASPA,TYMS,TF,MOBP,DYSF,ELOVL1,SH3TC2,ADAMTS1,C21ORF91,MAG,S1PR5,LDB3,CAPN3,NKX6-2,FA2H,CNTN2,PIP4K2A,P2RX7,ATF3,DOCK10,TMEM98,CD9,CRYAB,USH1C,SEMA4D,TNC,NDE1,CERS2,MYO1D,TGFA,GAL3ST1,ABCA2,PLLP,ZNF536,RASSF2,SEMA6A,BOK,SYNJ2,SEMA3B,PHLDB1,MAN2A1,CLIC4,CFL2,BCAS1,DAAM2,KIF13B,WHRN,GLIPR2,FGF1,MCAM,FRYL,PLAAT3,SFRP1,SOX10,MAP4K4,COL5A2,LTK,CARTPT
GO:BP	Glycosylceramide biosynthetic process	GO:0046476	0.019890103	1.70136298	8	105	3	17906	UGT8,FA2H,GAL3ST1
GO:BP	Central nervous system development	GO:0007417	0.022798319	1.64209718	1031	158	24	17906	LRP2,AQP1,SLC45A3,MOG,PLP1,KLK6,UGT8,ASPA,C21ORF91,MAG,NKX6-2,FA2H,CNTN2,TMEM98,CD9,NDE1,MYO1D,ABCA2,BOK,SYNJ2,DAAM2,WHRN,SFRP1,SOX10
GO:BP	Lipid biosynthetic process	GO:0008610	0.023671623	1.62577197	711	155	19	17906	SLC45A3,PLP1,UGT8,ELOVL1,PLPP2,FA2H,SLC44A1,PIP4K2A,P2RX7,CYB5R2,CERS2,PLD1,GAL3ST1,ABCA2,ACSL1,SYNJ2,GPD1,FGF1,PLAAT3
GO:BP	Regulation of cell development	GO:0060284	0.027719447	1.55721544	991	166	24	17906	LRP2,SLC45A3,KLK6,SPP1,ASPA,DOCK5,C21ORF91,MAG,S1PR5,NKX6-2,CNTN2,TMEM98,SEMA4D,CERS2,ZNF536,SEMA6A,SEMA3B,MAN2A1,DAAM2,KIF13B,SFRP1,SOX10,MAP4K4,LTK
GO:BP	Positive regulation of bleb assembly	GO:1904172	0.028707127	1.54201028	2	71	2	17906	ANLN,P2RX7
GO:BP	Regulation of bleb assembly	GO:1904170	0.028707127	1.54201028	2	71	2	17906	ANLN,P2RX7
GO:BP	Galactosylceramide metabolic process	GO:0006681	0.029707822	1.52712919	9	105	3	17906	UGT8,FA2H,GAL3ST1
GO:BP	Negative regulation of cell development	GO:0010721	0.029857665	1.52494416	350	160	13	17906	SPP1,MAG,NKX6-2,CNTN2,TMEM98,SEMA4D,CERS2,ZNF536,SEMA6A,SEMA3B,DAAM2,SOX10,MAP4K4
GO:BP	Drug transmembrane transport	GO:0006855	0.032057254	1.49407368	75	2	2	17906	LRP2,AQP1
GO:BP	Generation of neurons	GO:0048699	0.033131105	1.47976408	1574	166	32	17906	LRP2,SLC45A3,PLP1,KLK6,UGT8,SPP1,GLDN,ASPA,C21ORF91,MAG,S1PR5,NKX6-2,CNTN2,DOCK10,TMEM98,USH1C,SEMA4D,TNC,NDE1,CERS2,ZNF536,SEMA6A,SEMA3B,MAN2A1,DAAM2,KIF13B,WHRN,FRYL,SFRP1,SOX10,MAP4K4,LTK
GO:BP	Ceramide metabolic process	GO:0006672	0.034955601	1.45648322	94	105	6	17906	UGT8,ELOVL1,FA2H,P2RX7,CERS2,GAL3ST1
GO:BP	Transmembrane transport	GO:0055085	0.038251324	1.41735353	1634	73	19	17906	LRP2,AQP1,PIEZO2,KCNH8,SLC45A3,ABCA8,TMEM144,GJB1,TF,SGK2,TMEM63A,DYSF,SLC5A11,CLCA4,TTYH2,CAPN3,SLC44A1,P2RX7,SLC26A9
GO:BP	Cellular component assembly involved in morphogenesis	GO:0010927	0.039919771	1.39881196	114	132	7	17906	UGT8,LDB3,CAPN3,CD9,ABCA2,PHLDB1,CFL2
GO:BP	Galactolipid metabolic process	GO:0019374	0.042258663	1.37408425	10	105	3	17906	UGT8,FA2H,GAL3ST1
GO:BP	Regulation of plasma membrane bounded cell projection organization	GO:0120035	0.045082781	1.3459893	694	166	19	17906	ANLN,KLK6,ENPP2,SPP1,C21ORF91,MAG,CNTN2,P2RX7,USH1C,SEMA4D,CERS2,PLD1,CDC42EP1,SEMA6A,SEMA3B,KIF13B,SFRP1,MAP4K4,LTK
GO:BP	Regulation of cellular component size	GO:0032535	0.049709667	1.30355914	383	132	12	17906	AQP1,SPP1,MAG,CNTN2,P2RX7,USH1C,SEMA4D,WIPF1,CDC42EP1,SEMA6A,SEMA3B,CFL2

Various genes i.e., *DAAM2, CERS2, SLC45A3, PLP1, ASPA, SH3TC2, MAG, FA2H, CNTN2, TMEM98, ABCA2, CD9, BOK, SOX10, NKX6-2* were also involved in gliogenesis, glial cell development and differentiation as evident by pathway enrichment analysis. Pathway enrichment analysis also demonstrated significant enrichment for neurogenesis and nervous system development represented by *AQP1, MOG, PLP1, KLK6, UGT8, CLDN11, SPP1, GLDN, GJB1, ASPA, MOBP, SH3TC2, MAG, SEMA4D, SEMA6A, SEMA3B, TNC, SOX10, MAP4K4, UGT8.*

**Fig. 5:**
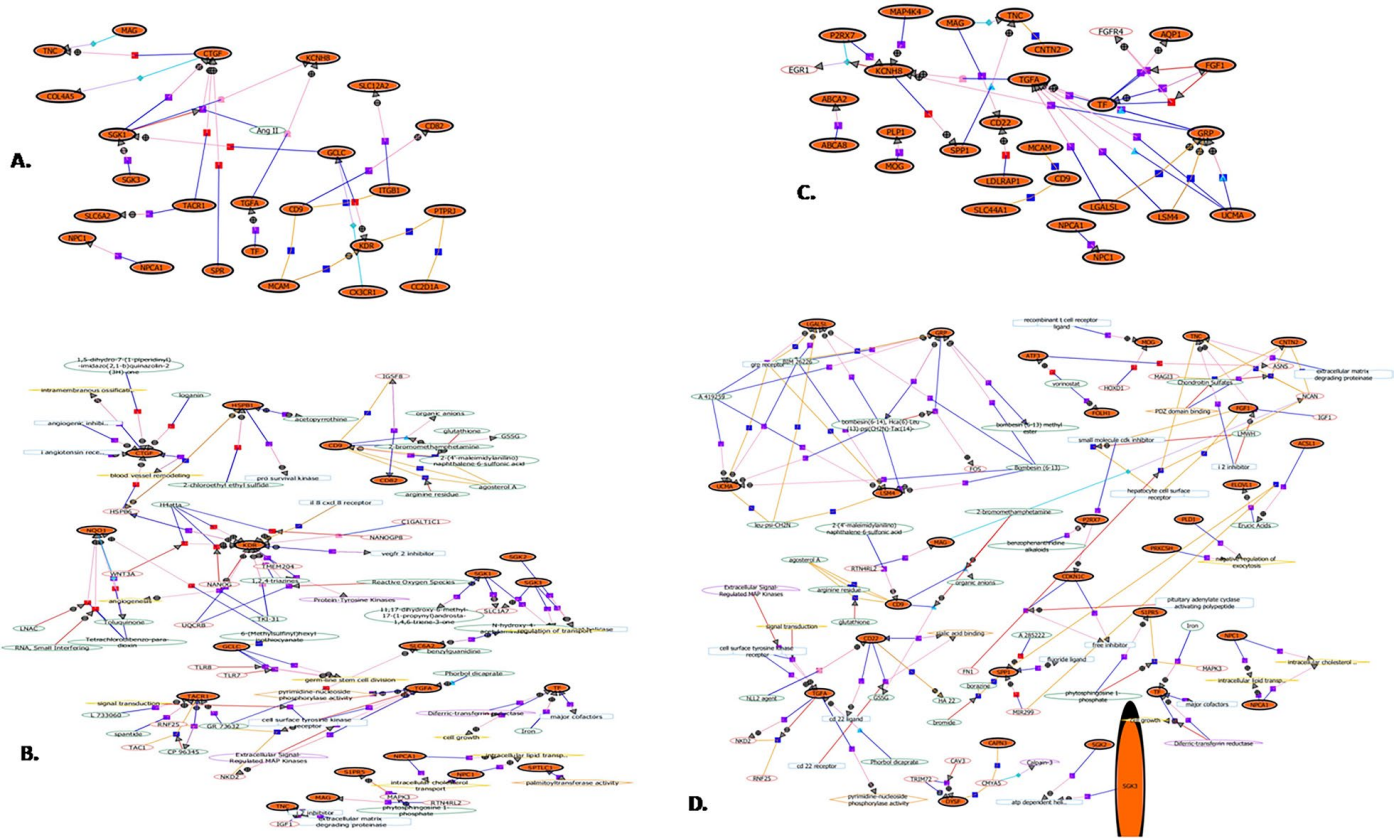
Gene network analysis showing associations between significantly modulated DEGs: **A** Direct interactions for MAX vs autopsy control **B** Indirect interactions for MAX vs autopsy control. **C** Direct interactions for MAX vs MIN **D** Indirect interactions for MAX vs MIN. Genes found modulated in our study are shown in filled circles. Different coloured edges with arrows shows the direction of interactions. Details of the graphical display of these associations are provided in Additional file 2: Fig. S2

Network analysis also revealed the interaction of various genes including several of above-mentioned genes. Network analysis demonstrated associations of *CTGF, MAG, TNC, SLC12A2, SLC6A2, SGK1, SPTLC1, NPC1* and *TF* in MAX vs autopsy and *MAP4K4, CNTN2, P2RX7, KCNH8, TNC, GRP, FGF1, TGFA, MAG, PLP1, PLD1, ABCA2, ABCA8, AQP1, ATF3, ELOVL1, MOG* and *NPC* in MAX vs MIN, further strengthen their role in pathophysiology of FCD type II (Fig. 5).

### Validation of data by real-time PCR

**Fig. 6:**
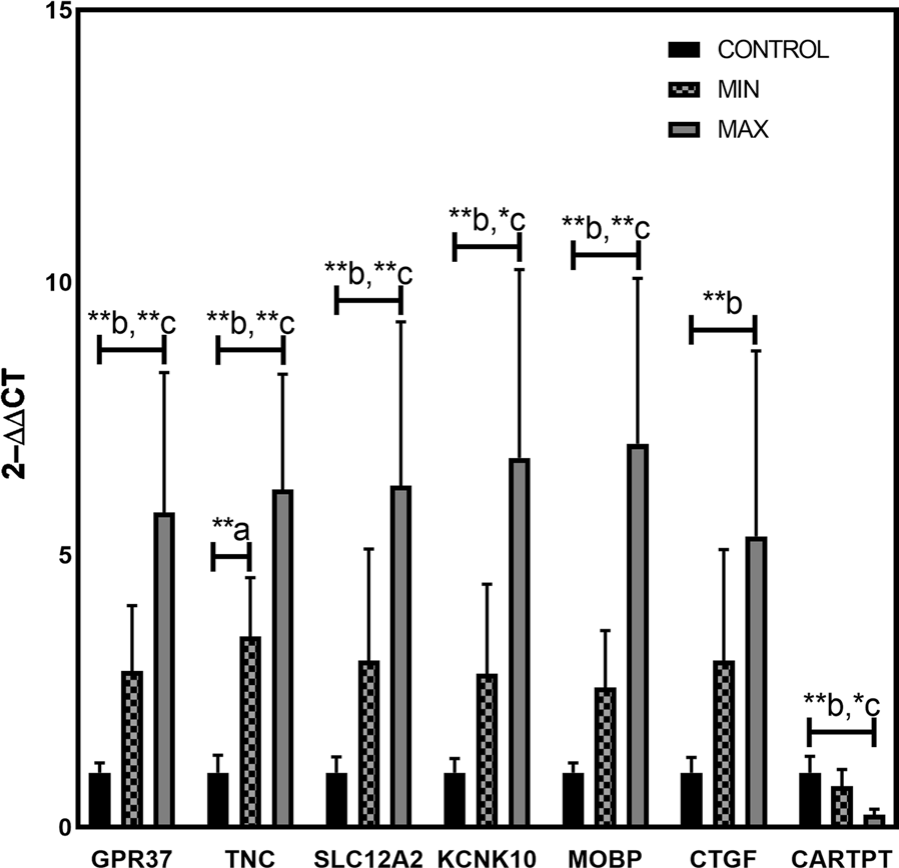
Real-time PCR validation of selected DEGs. The expression level of up-regulated mRNAs (TNC, MOBP, CTGF, SLC12A2, GPR37, and KCNK10) and down-regulated mRNA (CARTPT) as identified in the RNAseq data were further validated by real-time PCR. Relative changes in gene expression were calculated using the ΔΔCq method with HPRT as a reference gene. Error bar is mean ± SD based on nine patients and eight control samples, and each sample is analyzed in triplicates. Mean increase in transcripts levels are statistically significant (One-way ANOVA followed by the Tukeys’ post hoc test; *p < 0.05; **p < 0.01; a = MIN vs autopsy, b = MAX vs autopsy; c = MAX vs MIN)

The mRNA levels of *TNC, SLC12A2, CTGF, KCNK10, MOBP,* and *GPR37* were significantly up-regulated in MAX compared to autopsy controls (fold-change ≥ 2; p value < 0.05), whereas *CARTPT* was down-regulated (fold-change ≥ 2; p value < 0.05) (Fig. 6). The mRNA levels of *TNC, KCNK10, MOBP, SLC12A2* and *GPR37,* were significantly up-regulated in MAX compared to MIN (fold-change ≥ 2; p value < 0.05), whereas *CARTPT* was significantly down-regulated. *CTGF* expression was relatively higher in MAX as compare to MIN, but it was not statistically significant (Fig. 6). Only *TNC* expression was significantly higher in MIN as compare to autopsy (fold-change ≥ 2; p value < 0.05) (Fig. 6).

## Discussion

In the present study, we utilized ECoG-graded clinically well-characterized brain tissue resected during surgical treatment of drug-resistant FCD type II patients to evaluate all elements of the transcriptome towards developing a better understanding of the molecular mechanisms underlying the pathogenesis of FCD type II with the ultimate goal of identifying novel markers to help to localize EZ. Only a few high throughput profiling studies have been conducted on focal cortical dysplasia patients [15, 17–20]. Integrated genome-wide DNA methylation and RNAseq analysis identifies aberrant signalling pathways related to receptor tyrosine kinases (RTK), EGFR, PDGFRA in FCD type II patients [15]. Contrary to our results, transcriptomic profiling of dysplastic human temporal neocortex demonstrated the down-regulation of myelination-associated transcripts [17]. Other transcriptomic studies compared acutely high-spiking cortical areas against non-spiking cortical areas to look at seizure-induced gene expression [19, 20]. Similar to our results, Arion et al*.* (2006) demonstrated up-regulation of myelination associated genes. They also demonstrated the down-regulation of multiple GABA system-related genes (*GABRA5, GABRB3, ABAT*) and alterations in transcripts related to various signalling cascades in the spiking samples from temporal lobe patients [19], but we did not observe any alterations in GABA related gene expression. Similarly, Dachet et al*.* (2015) compared acutely high-spiking versus non-spiking cortical areas in neocortical epilepsy patients. They demonstrated increased expression of genes related to endothelial, red and white blood cells, neurons, and microglia, but a decreased expression of oligodendrocyte-specific transcripts in high spiking cortical regions [20]. Here, we are discussing the role of the DEGs and their possible association with epileptogenesis in FCD type II by grouping them into specific pathways.

### Myelination, axon and neuronal ensheathment, oligodendrocyte development and differentiation

Oligodendrocyte-specific and myelination-associated genes were one of the dominating functional groups found to be up-regulated in MAX of dysplastic tissues in FCD type II patients compared to MIN and autopsy control. These include *PLP1, SOX10, MAG, MOG, MOBP, KLK6, UGT8, CLDN11, ASPA, SH3TC2, NKX6-2, CTGF, SEPT 4, CDKN1C, GLDN, SPP1, CNTN2, TMEM98, TMEM10 (OPALIN), ANLN, ERMN, ENPP2, CD9, ABCA2, ABCA8, SPP1* and *TF.*

*PLP1, MAG, MOG, MOBP, TMEM10 (OPALIN), ASPA, ABCA2, TF, GLDN, and SOX10* all play important roles in the oligodendrocytes (OLs) differentiation and myelination [30–37]. *KLK6,* a serine protease, may rapidly hydrolyze major myelin and blood brain barrier proteins and promote oligodendrogliopathy, neuronal injury and astrogliosis [38, 39]. *ANLN* from oligodendrocytes disrupts myelin septin assembly, causing the appearance of abnormal myelin outfoldings*. ERMN* plays a significant role in cytoskeletal rearrangements during the late wrapping and/or compaction phases of myelin assembly [40, 41]. Contrary to this study, many of these genes, *GLDN, MOBP, UGT8, ASPA, TMEM10 (OPALIN), MOG, ERMN,* and *CLDN11* were found to be down-regulated in dysplastic human temporal neocortex [17]. Similar to this study, increased expression of *MOG, PLP1, ABCA2, FA2H, TF, ASPA* was demonstrated in high-spiking regions of cortical areas of temporal lobe epilepsy patients [19].

ECM related genes, i.e., *TJP2, CLDN9, CLDN11, SPP1,* and *GJB1 (CX32),* were found to be up-regulated in the MAX region of FCD patients. Lee et al. (2007) demonstrated the up-regulation of *UGT8, MOG, TJP2, and ENPP2* in temporal lobe epilepsy patients [42]. These molecules are important for maintaining the proper physiological ambience for the timely development of oligodendrocyte precursor cells (OPCs) into myelinating OLs [43–45]. *SH3TC2/KIAA1985, ABCA2* and *ABCA8* are supposed to be involved in cargo transport for myelin formation [46, 47]. *GPR37* (G protein-coupled receptor 37) negatively regulates oligodendrocyte differentiation and myelination [48]. Likewise, increased expression of *GPR37* was reported in the high-spiking region of temporal lobe epilepsy patients [19]. A mutation in  *CNTN2* may be associated with adult myoclonic epilepsy [49].

*CTGF* expression was found to be up-regulated in MAX of surgically resected sample of patients compared to autopsy controls. *CTGF/CCN2* negatively regulates myelination through the mTOR pathway [50]. Mutations in mTOR pathway genes were reported in FCD [51, 52]. Our previous study also demonstrated differential epigenetic regulation of the mTOR pathway in FCD [15]. Overtly active mTOR signaling may lead to insufficient myelination associated with FCD type II. *CTGF* has also been linked to astrogenesis, astrocyte activation, and neuro-inflammation [53].

In the present study, we have demonstrated the increased expression of genes related to myelination, remyelination or demyelination, suggesting that both phenomena are prevalent in patients. Demyelination is compensated for by remyelinating factors, and a delicate balance between them must be disrupted, resulting in myelin pathology, which may contribute to the epileptogenicity of this cortical malformation. OLs' inability to synthesize functional myelin could also be a factor. Up-regulated expression of several OL differentiation related genes could be due to an increased number of OPCs and differentiating OLs. It could be due to a compensatory mechanism to suppress epileptiform activity. Reductions in the number of oligodendroglial cells and myelin content have been reported in FCD, but the results remain controversial. An increased number of oligodendendroglia was also reported in patients with temporal lobe epilepsy and malformations of cortical development [54–57]. Scholl et al. (2017) suggested that impaired oligodendroglial turnover is associated with myelin pathology in focal cortical dysplasia and tuberous sclerosis complex. Proliferative oligodendroglia was identified in FCD IIA, IIB, and TSC, suggestive of a reactive phenomenon due to insufficient maturation or delayed maturation that prevents adequate myelination [55].

Recent studies show that neuronal activity can influence the generation of new oligodendrocytes (oligodendrogenesis) and myelination. Changes in myelination in cortical white matter are mostly reported, but alterations in myelination of grey matter have also been demonstrated [58]. During epileptogenesis, various kinds of synchronous sub-threshold excitatory stimuli allow their temporal summation in the post synaptic neurons [59]. This summation could be a direct result of axons with poorly distributed conduction velocities, resulting in synchronous action potential firing. The conduction velocity of an axon is mainly related to its diameter and the myelin sheath. Therefore, a direct relation might exist between epileptic seizure susceptibility and abnormal myelin content. Conversely, previous studies have indicated that neurological disorders associated with abnormal myelin content are accompanied by a higher susceptibility to epileptic seizures [60–63]. Several studies have indicated that epilepsy is also associated with myelin abnormalities [64–69]. Oligodendrocytes also control potassium accumulation in white matter and seizure susceptibility [70]. A subset of CNS oligodendrocytes expresses glutamine synthetase and directly modulates glutamatergic excitatory neurotransmission [71]. The findings presented here highlight avenues for potential therapeutic interventions targeting aberrant oligodendrogenesis and myelination.

### Phospholipid biosynthesis

RNA Seq data highlights the perturbation of key metabolism processes in lipid metabolism, especially phospholipid biosynthesis in the MAX region of the FCD type II patients. Altered lipid levels and/or distribution have been reported in a variety of neurodegenerative diseases. [43, 72, 73]. *PLLP, UGT8, ABCA2, PLD1, ELOVL1, CERS2, S1PR5, PLPPR1, SPTLC1, ENPP2, NPC1, FA2H, LRP2, S1PR5* and *GAL3ST1* gene expression were found to be up-regulated in this study. *PLLP, CERS2, UGT8, ASPA* and *GAL3ST1* contribute to various processes related to myelin synthesis [74]. *PLD1, ELOVL1, NPC1, SPTLC1, FA2H, LRP2, and S1PR5* contribute to the synthesis of fatty acids, sphingolipids, and intracellular trafficking of lipid molecules [75–77]. Our data demonstrated dysregulation in lipid metabolism, i.e. phospholipid biosynthesis and trafficking, which in turn ameliorates the signalling pathways related to lipid molecules and can affect diverse cellular functions. Apart from these, phospholipids are known to be important regulators of many channels, mitochondrial function, excitotoxicity, impaired neuronal transport, cytoskeletal defects, inflammation, and reduced neurotransmitter release [72]. Future studies on these altered genes could provide us with promising targets with the potential to delineate the epileptogenic zone in FCD type II.

### Ion channels

Ion channel dysfunction, either caused by mutations or acquired, has been associated with epilepsy. Many AEDs tend to manipulate the ion permeability of these channels to modify neuronal excitability [78]. In the present study, we have demonstrated the up-regulation of *AQP1, KCNK10, KCNH8, P2RX7, SGK1, SGK2, SLC12A2, SLC6A2, SLC44A1, SLC45A3, SCLC5A11, SCL26A9, CLCA4* and *SEPT4* in MAX of FCD type II patients.

*AQP1* functions as a water channel protein, whereas KCNK10 and KCNH8 are potassium channels for neurotransmitter release, neuronal excitability, and electrolyte transport [79, 80]. *SGK1* and *SGK2* are reported to be involved in the regulation of a wide variety of ion channels, i.e., potassium, sodium, and chloride channels, membrane transporters, cell growth, survival and proliferation [81]. Activation of the *P2X7* receptor has been associated with neuronal excitability, microglia activation and neuro-inflammatory responses. Increased expression of the *P2X7* receptor has been demonstrated in animal models of epilepsy. *P2X7* receptor ligands may be considered as a therapeutic target for DRE [82]. High *SLC12A2* expression results in elevated Cl- concentration inside the cell, leading to net Cl^−^ outflow and subsequent depolarization when GABA activates GABA_A_Rs [83, 84]. Increased expression of *SLC12A2* has been reported in surgically resected tissue specimens from FCD patients [85]. *SLC26A9*, a highly selective chloride ion channel, *CLCA4*, calcium sensitive chloride channel, *SLC45A3*, *SLC5A11* may be involved in ion transport and neurotransmitter release in FCD [78]. *SLC44A1,* a choline transporter, may contribute to membrane synthesis and myelin production. Alterations in ion channel gene expression might affect the ionic homeostasis of ions involved in epileptic activity within dysplastic tissues. So, it could serve as a potential biomarker to identify the EZ in FCD patients, but confirmatory studies on a larger cohort are needed.

### Cell signaling molecules of various functions

Aside from these, several genes related to diverse cellular functions were found to be altered in this study, including semaphorins, fibroblast growth factors (FGFs), *MAP4K4, ATF3, TNC, CALB2* and *GRP*. Here, we demonstrated the up-regulation of *SEMA3B, SEMA4D* and *SEMA6A* in MAX compared to MIN. *SEMA3B-NRP1* mediated immune response and apoptosis have been reported, and their involvement in neuro-inflammation and cell death in epileptic conditions cannot be ruled out [86, 87]. *SEMA4D/CD100* may regulate oligodendrocyte differentiation by promoting apoptosis [88]. *SEMA4D* also promotes inhibitory synapse formation and alleviates seizures in an animal model of epilepsy [89]. *SEMA6A* is considered to be a positive regulator of oligodendrocyte differentiation and myelination [90].

Other than semaphorins, the expression of *MAP4K4, TNC, FGF1, FGF17, TGFA, ATF3, MCAM* was found to be up-regulated and *GRP* and *CALB2* were down-regulated in the study. *MAP4K4* plays a specific role in activating the MAPK8/JNK pathway, which has also been found to be up-regulated in high-spiking cortical areas of TLE patients [19, 91]. Increased expression of *TNC* is highly associated with glial reactivity and reduced myelination, and also participates in Notch signalling [92]. As the *FGF* system is involved in the development of specific brain circuits in the hippocampus and cortex associated with epileptogenesis, increased expression of *FGF1* and *FGF17* was very much expected. *FGF17* can activate numerous transcription factors involved in intra-cortical wiring. FGF1 has also been linked to a role in an animal model of epilepsy. Contrary to this, FGF1 has been shown to have anti-convulsant properties in kainate-induced epilepsy [93]. *ATF-3* expression has been correlated with seizure frequency in epilepsy patients [94]. Loss of *CALB2* (Calretinin) expression in hippocampal interneurons was shown in the dentate gyrus of patients with epilepsy [95]. Contrary to this, an increase in the number of calretinin-positive cells was observed by Blumcke et al. (1999) in patients with temporal lobe epilepsy [96]. Further studies on a greater number of samples are required for absolute findings.

There is evidence that *GRP* mediated signalling might play a role in regulating cognitive functions such as emotional responses, social interaction, memory, and feeding behaviour. Alterations in *GRP* or *GRPR* expression or function have been reported in patients with neurodegenerative, neurodevelopmental, and psychiatric disorders [97].

The small sample size of this study which does not include age and gender matched cases and controls is one of its limitations. Only two autopsy samples have been included in the present study. The age range of FCD patients is from 5 to 22 years, whereas autopsy patients range from 16 to 65 years. It’s very difficult to obtain age and gender-matched autopsy samples as per the inclusion criteria. Surgically resected tissue samples obtained for this study were from patients suffering from seizures for many years. Therefore, it is difficult to delineate and relate the transcriptional changes to underlying epileptogenic changes and to seizure activity. Further in vitro and in vivo studies are needed to determine whether the identified transcriptional changes are epileptogenic or a symptom of seizure activity.

The patients with FCD were on a combination of anti-epileptic drugs which may affect the expression of certain genes. AEDs selectively reduce the excitability of neurons and provide appropriate seizure control in epileptic patients by acting on a variety of biological targets. AEDs have a variety of modes of action, which can be classified based on their regulatory roles in voltage-gated ion channels and synaptic excitability control. However, recent research has revealed that AEDs can act as epigenetic modifiers to regulate gene expression [98]. Changes in gene expression caused by Valproate were seen in the peripheral blood of patients with newly diagnosed epilepsy [99]. The antiepileptic drug levetiracetam selectively modifies kindling-induced alterations in gene expression in the temporal lobe of rats [100]. These studies suggest that AEDs may have modulatory effects on the expression of certain genes. Hence, the contribution of AEDs to changes in gene expression cannot be ruled out. The findings of this study suggest that myelin and/or oligodendrocyte cells are involved in the epileptogenic process. Further exploration of the altered pathways may provide potential markers to aid in specifying the EZ in FCD patients. To date, there have been several preclinical and human studies presenting clear evidence that myelin content could be associated with epilepsy, epileptic seizures and epileptogenesis. Attempts to restore the process of myelination through pharmacological intervention could represent another promising therapeutic strategy for FCD as there is no evidence that administering these drugs to human patients can prevent seizures [58, 62]. Even with potential limitations, our study shows a tight association between ECoG grading of samples and the expression pattern of *PLP1, PLLP, UGT8, KLK6, SOX10, MOG, MAG, MOBP, ANLN, ERMN, SPP1, TF, FA2H, CLDN11, TNC, GPR37, GRP, ABCA2, ABCA8, ASPA, P2RX7 (P2X7), CERS2, MAP4K4, OPALIN, Semaphorins, FGF1, CALB2,* and *TNC* in patients with FCD. These genes could be further studied as a potential biomarker for the identification of epileptogenic margins in these patients. The primary reason for poor surgical outcomes in patients with FCD is the inaccurate localization of the epileptogenic margins. These results further support that EcoG-guided resection is likely to have a better outcome in terms of achieving seizure freedom post-operatively [22, 101].

## Supplementary Information

**Additional file 1: Figure S1.** Representative image of EcoG recording of a 26-year-old male patient showing MAX and MIN region.

**Additional file 2: Figure S2.** Symbols used in network analysis.

**Additional file 3.** List of significantly altered genes analyzed by CuffDiff.

**Additional file 4.** List of significantly altered genes analyzed by DESeq 2.

**Additional file 5.** List of significantly altered genes analyzed by EdgeR.

**Additional file 6.** List of common genes found  significantly altered in all three  softwares.

**Additional file 7: Table S1.** List of primers used in the study.

## Data Availability

RNAseq data and sequences are submitted at NCBI BioProject (http://www.ncbi.nlm.nih.gov/bioproject/PRJNA369732).

## Abbreviations

AEDs: Anti-epileptic drugs
ALS: Amyotrophic lateral sclerosis
DEGs: Differentially expressed genes
DRE: Drug-resistant epilepsy
EZ: Epileptogenic zone
ECOG: Electrocorticography
EEG: Electroencephalogram
FCD: Focal cortical dysplasia
MCD: Malformation of cortical development
MSA: Multiple system atrophy
OLs: Oligodendrocytes
OPCs: Oligodendrocytes precursor cells
RNAseq: RNA sequencing
MRI: Magnetic resonance imaging
PCA: Principal component analysis
PET: Positron emission tomography
MEG: Magnetoencephalography
GO: Gene ontology
