# Supplementary material for: Transcriptomic profiling of high- and low-spiking regions reveals novel epileptogenic mechanisms in focal cortical dysplasia type II patients
Source: Mol Brain. 2021 Jul 23;14:120. doi: 10.1186/s13041-021-00832-4 (PMC8305866; doi:10.1186/s13041-021-00832-4)
Supplement: Supplementary file 1 — Additional file 1: Figure S1. Representative image of EcoG recording of a 26-year-old male patient showing MAX and MIN region. [file 13041_2021_832_MOESM1_ESM.pptx]

## Slide 1
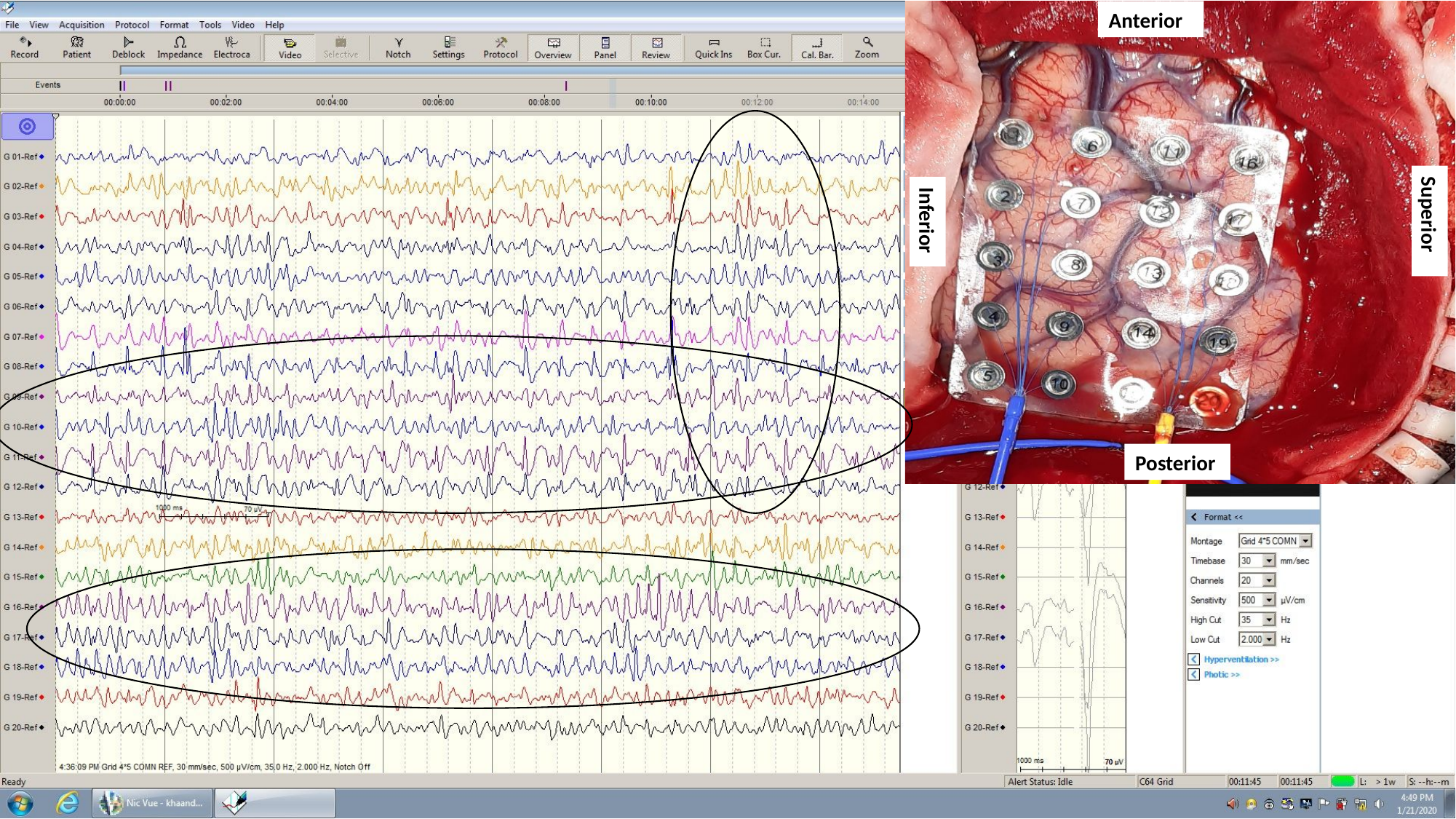

Anterior
Superior
Inferior
Posterior

## Slide 2
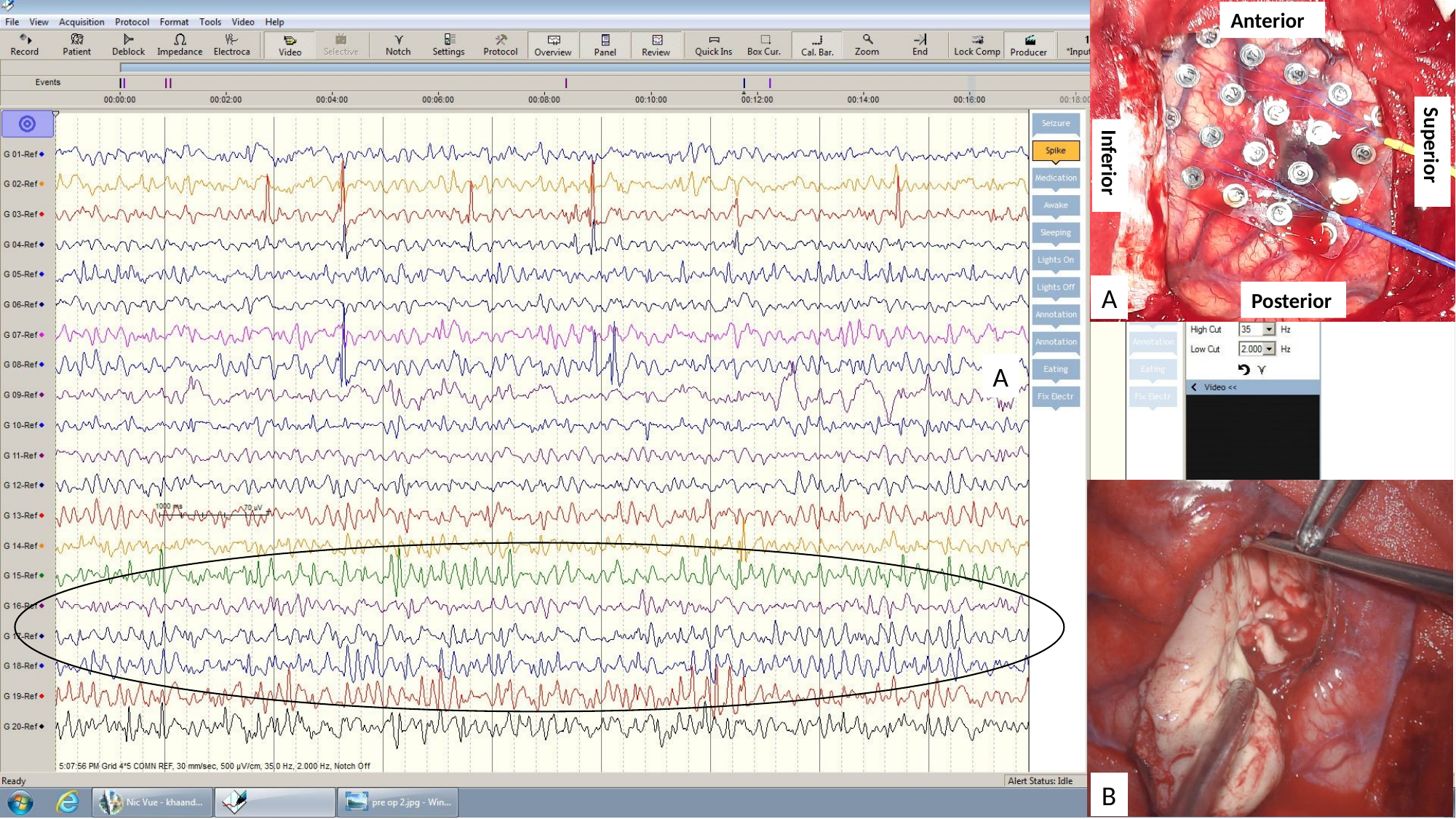

Anterior
Superior
Inferior
Posterior
A
A
B

## Slide 3
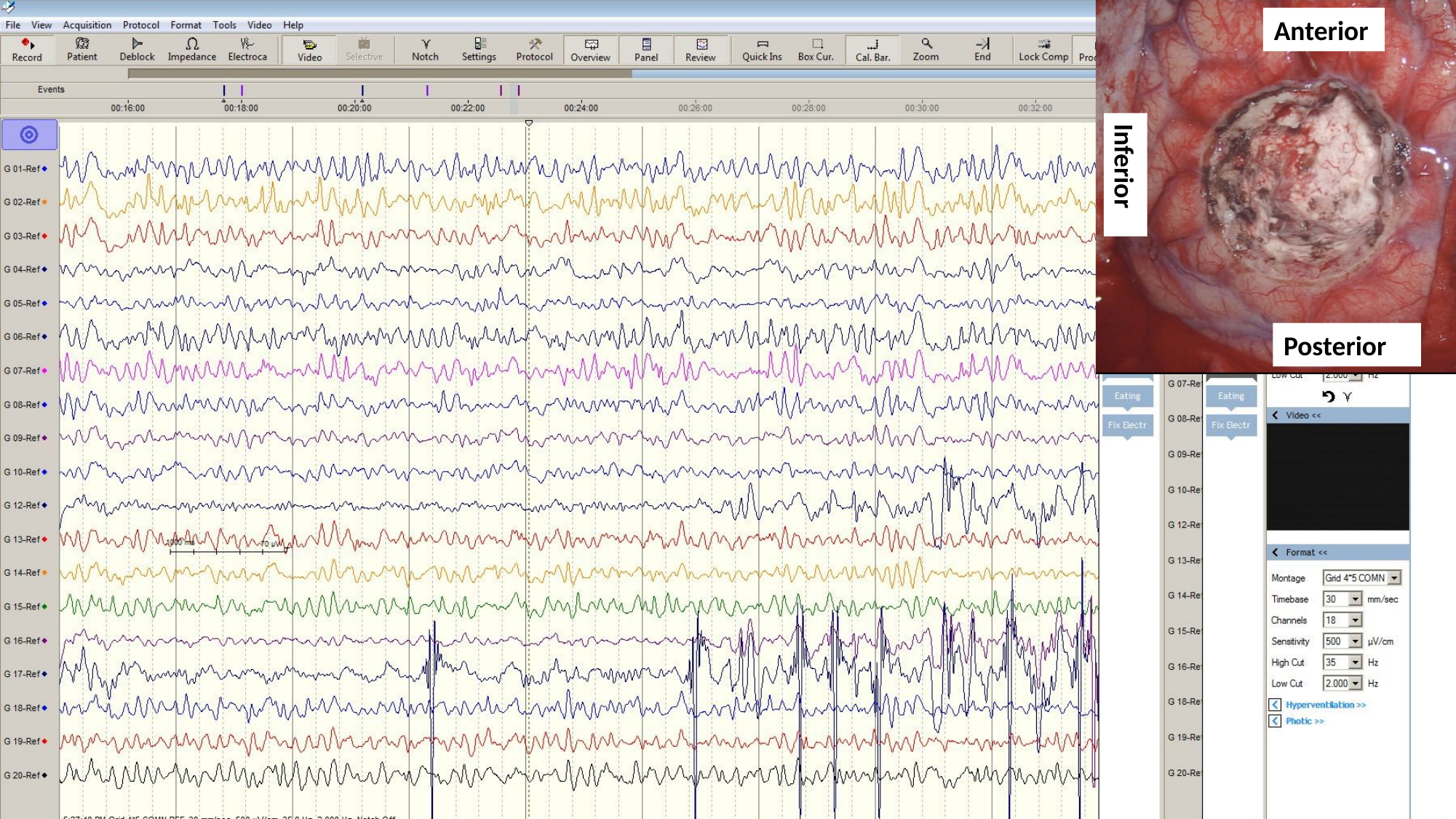

Anterior
Superior
Inferior
Posterior
