# Supplementary material for: Transcriptomic profiling of high- and low-spiking regions reveals novel epileptogenic mechanisms in focal cortical dysplasia type II patients
Source: Mol Brain. 2021 Jul 23;14:120. doi: 10.1186/s13041-021-00832-4 (PMC8305866; doi:10.1186/s13041-021-00832-4)
Supplement: Supplementary file 2 — Additional file 2: Figure S2. Symbols used in network analysis. [file 13041_2021_832_MOESM2_ESM.docx]

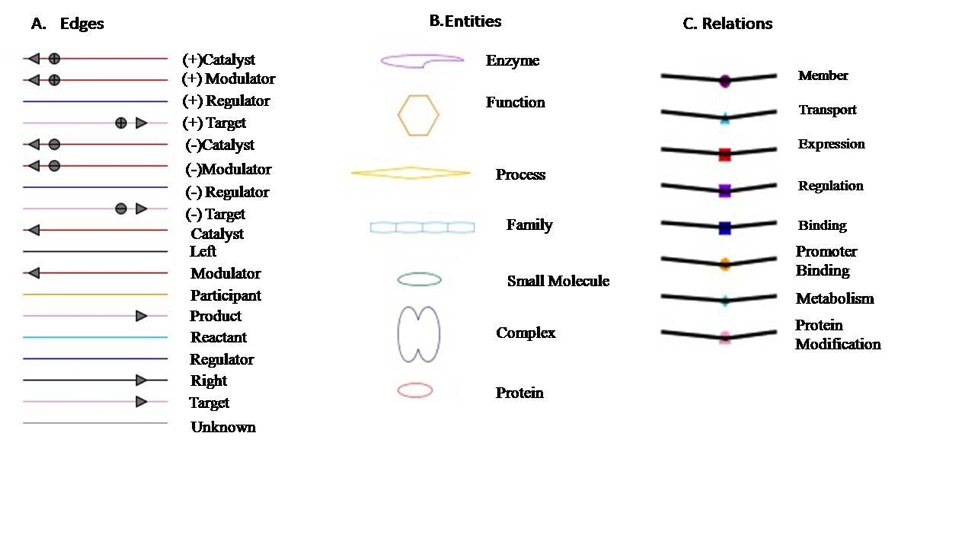


**Figure S2: Graphical display of the various type of associations between different epileptic genes.(A)** Different colored edges with arrows shows the direction of the interactions and circles with + and – symbols represent positive and negative regulation. **(B)**Various entities not reported in our study but are associated with the epileptic genes (66) are enclosed in different colored shapes. **(C)** Various edges and symbols (small squares, triangles and circles) shows diiferent modes of regulations.
