# Supplementary material for: Transcriptomic profiling of high- and low-spiking regions reveals novel epileptogenic mechanisms in focal cortical dysplasia type II patients
Source: Mol Brain. 2021 Jul 23;14:120. doi: 10.1186/s13041-021-00832-4 (PMC8305866; doi:10.1186/s13041-021-00832-4)
Supplement: Supplementary file 7 — Additional file 7: Table S1. List of primers used in the study. [file 13041_2021_832_MOESM7_ESM.docx]

| Gene | Primers |
| --- | --- |
| TNC | F - GTCACCGTGTCAACCTGATG |
|  | R-GCCTGCCTTCAAGATTTCTG |
| SLC12A2 | F - AATGATGGCCACTGTTGTGA |
|  | R - TAGACCAATTGCACCACCAA |
| CTGF | F - GGAAAAGATTCCCACCCAAT |
|  | R - TGCTCCTAAAGCCACACCTT |
| KCNK10 | F - TGTCCCTTGGGTGTCTTAGG |
|  | R - CGCTGCAAGCAATAAAAACA |
| MOBP | F - GGTAGGGTGAGCTGGTGTGT |
|  | R -AACAAAAGGCCCTCAAGGTT |
| GPR37 | F- ACGGTGACCAGTGATGACAA |
|  | R- CAGCAATGAGTTCCGACAGA |
| CARTPT | F- ACGAGAAGGAGCTGATCGAA |
|  | R- TTCCTCACTGCACACTGCTC |
| HPRT | F-GCCCCATCTGTGTTGATTCT |
|  | R-GGTCCTTTTCACCAGCAAGCT |

Table S1. Real time PCR primers
